# High-yield cell-derived extracellular matrix bioink via macromolecular crowding for versatile 3D bioprinting

**DOI:** 10.1016/j.mtbio.2026.103135

**Published:** 2026-04-17

**Authors:** Siwi Setya Utami, Honghyun Park, Jueun Kim, Aram Sung, Min-Ju Choi, Ga-Hee Ryoo, Chang Woo Gal, Insup Kim, Hui-Suk Yun, Yeong-Jin Choi

**Affiliations:** a Advanced Materials Engineering, Korea National University of Science and Technology (UST), 217, Gajeong-ro, Yuseong-gu, Daejeon, 34113, Republic of Korea; b Advanced Bio and Healthcare Materials Research Division, Korea Institute of Materials Science (KIMS), 797, Changwon-daero, Seongsan-gu, Changwon, 51508, Republic of Korea

**Keywords:** Cell-derived extracellular matrix (CD-ECM), Macromolecular crowding (MMC), Bioink, Bone tissue engineering, Bone tissue regeneration

## Abstract

Decellularized extracellular matrix (dECM) is a promising bioink because it replicates the biochemical and structural features of native tissues. However, tissue-derived dECM is limited by restricted availability and potential immunogenicity. To overcome these challenges, we developed a macromolecular crowding (MMC)-enhanced cell-derived extracellular matrix (CD-ECM) bioink with improved yield and biofunctionality. MC3T3-E1 pre-osteoblasts were cultured under MMC conditions, followed by decellularization and enzymatic processing to generate a printable CD-ECM bioink. The MMC strategy markedly increased extracellular matrix (ECM) yield, collagen and glycosaminoglycan (GAG) content, and mechanical stability compared to conventional cultures. The optimized CD-ECM bioink exhibited reliable printability in both extrusion-based and digital light processing (DLP) 3D bioprinting, enabling fabrication of constructs with high shape fidelity and cell viability. Incorporation of α-tricalcium phosphate (α-TCP) further enhanced osteogenic performance, resulting in elevated alkaline phosphatase (ALP) activity, increased calcium deposition, and upregulation of osteogenic markers, including runt-related transcription factor 2 (RUNX2), collagen type I alpha 1(COL1A1), ALP, and osteocalcin (OCN). These findings highlight the synergistic interaction between ECM-derived biochemical cues and α-TCP-mediated ionic signaling. Overall, the MMC-enhanced CD-ECM/α-TCP bioink offers a versatile, biologically active, and osteoinductive platform for advanced bone tissue engineering and regenerative applications.

## Graphical abstract

**Figure:**
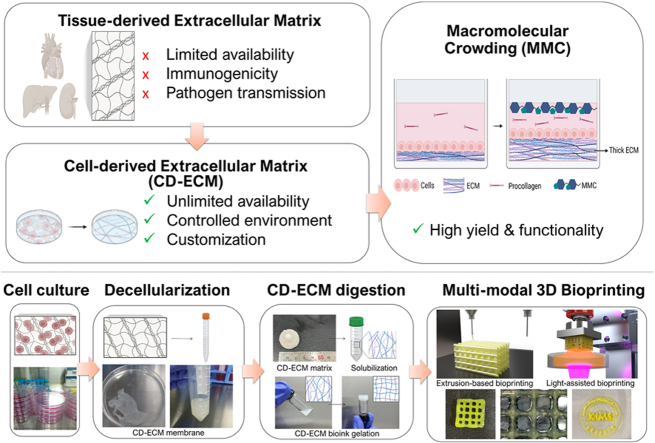

## 1 Introduction

Tissue engineering has advanced considerably with the development of biocompatible materials and 3D bioprinting technologies, enabling the fabrication of complex, cell-laden structures for regenerative medicine. Central to 3D bioprinting is the use of bioinks, which are printable, cell-laden hydrogels or biomaterial formulations designed to replicate native extracellular matrix and support tissue formation [1,2]. Among available bioinks, decellularized extracellular matrix (dECM)-based formulations have gained popularity due to their inherent biological signals and structural compatibility with host tissues. Studies have demonstrated that dECM bioinks enhance cell viability, guide lineage-specific differentiation, and promote functional tissue regeneration [[3], [4], [5]].

Despite these advantages, dECM-based bioinks face several challenges. Sourcing dECM from animal or human tissues raises concerns regarding limited availability, potential immunogenicity, pathogen transmission, and donor-to-donor variability [6]. Inconsistent biochemical composition, particularly in glycosaminoglycan (GAG) and collagen content, often results in batch-to-batch variability that compromises reproducibility. Furthermore, the use of non-human animal-derived tissue is associated with growing ethical concerns, amplified by recent regulatory shifts such as the United States (US) Food and Drug Administration (FDA) Modernization Act 2.0, which promotes alternative testing methods and reduces reliance on animal studies [7].

To address these limitations, cell-derived extracellular matrix (CD-ECM) has emerged as a promising alternative. CD-ECM is produced by culturing cells that secrete their own extracellular matrix (ECM) components, enabling a more standardized and ethically sustainable production process that does not rely on animal or human tissue [8,9]. Compared with non-human animal-derived dECM, CD-ECM offers improved control over biochemical composition, reduced donor variability, and greater batch-to-batch consistency, making it a sustainable approach. In our study, CD-ECM generated from MC3T3-E1 cells demonstrated reproducible GAG and collagen content across independent harvests, highlighting its potential for consistent and scalable bioink production.

However, a major limitation of CD-ECM is its inherently low yield, which restricts its utility in large-scale tissue engineering and 3D bioprinting [10,11]. Unlike conventional in vitro studies that require small amounts of ECM, 3D bioprinting aims to fabricate large, multilayered, and cell-laden constructs, demanding substantially higher volumes of ECM to prepare printable bioinks. To overcome this challenge, we adopted macromolecular crowding (MMC), which introduces inert macromolecules to the culture medium to recreate the densely packed in vivo environment. In such a crowded milieu, secreted ECM molecules (such as procollagen, fibronectin, and other matrix proteins) are confined to a reduced free volume because a significant fraction of the space is sterically occupied by crowders [12]. This “excluded volume” effect effectively increases the local concentration of ECM proteins and their processing enzymes, enhancing the frequency of productive collisions between them. As a consequence, procollagen is more efficiently processed, nascent collagen molecules more readily nucleate into fibrils, and fibrillar fibronectin networks are stabilized and expanded, providing scaffolding sites for further collagen deposition [13,14]. These coupled processes accelerate fibrillogenesis and supramolecular ECM assembly, leading to faster accumulation of matrix and the formation of denser, more organized collagen- and GAG-rich ECM layers around the cells [15,16]. Previous studies have shown that MMC stimulates ECM accumulation, promotes cell differentiation, and generates ECM-rich 3D microtissues [[16], [17], [18]]. However, its application in developing bioinks for 3D bioprinting has not yet been explored.

**Fig. 1:**
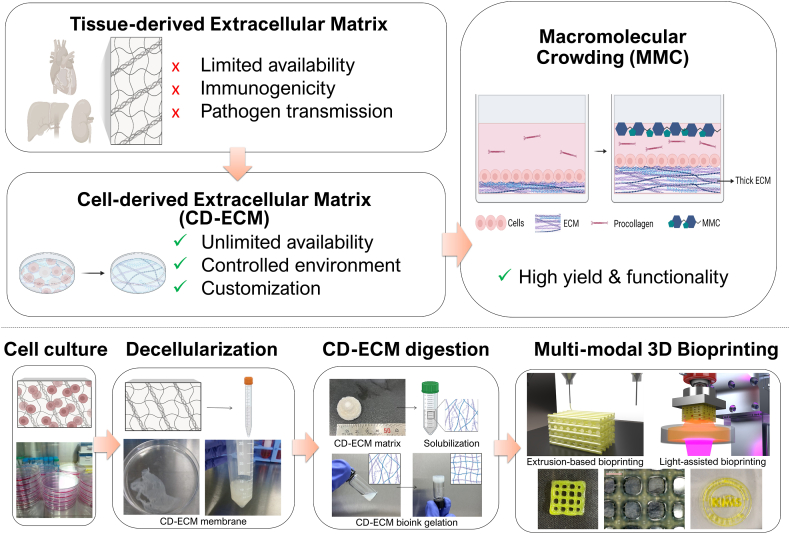
Schematic representation of cell-derived extracellular matrix (CD-ECM) bioink fabrication and applications.

In this study, we applied macromolecular crowding (MMC) to MC3T3-E1 pre-osteoblasts to develop a high-yield CD-ECM bioink tailored for bone tissue engineering and 3D bioprinting applications (see Fig. 1). MC3T3-E1 is a mouse calvaria-derived pre-osteoblast cell line that has been widely used as a representative osteogenic model due to its rapid proliferation and robust ECM production. Therefore, it was selected as a reproducible system for proof-of-concept optimization of the MMC-based CD-ECM production platform. While CD-ECM has been investigated in tissue engineering, its direct application as a bioink for 3D bioprinting remains limited, primarily due to the low ECM yield obtained under conventional culture conditions. To address this limitation, we established an MMC-enhanced CD-ECM production framework with defined culture conditions and controlled processing parameters, ECM yields sufficient for practical bioink fabrication. The resulting CD-ECM was processed into a bioink formulation and evaluated for compatibility with multiple bioprinting modalities, including extrusion-based and digital light processing (DLP)–based 3D bioprinting. In addition, incorporation of α-tricalcium phosphate (α-TCP) was explored to develop a composite ECM bioink with enhanced osteogenic functionality for bone tissue engineering. Importantly, MMC-mediated ECM enhancement was also confirmed in additional cell types, including C2C12 cells, human fibroblasts, and human adipose-derived stem cells (hADSCs), demonstrating that this platform is adaptable for generating tissue-specific and potentially fully human-derived CD-ECM bioinks. Overall, this study presents a scalable strategy for converting MMC-enhanced CD-ECM into a bioink-compatible material platform capable of supporting advanced biofabrication for bone regeneration.

## 2 Materials and methods

### 2.1 Decellularization optimization

#### 2.1.1 Optimization of macromolecular crowding conditions

The mouse calvaria-derived pre-osteoblast cell line MC3T3-E1 (passages 4–14, American Type Culture Collection, USA) was cultured in 6-well plates at a seeding density of 2 × 10^4^ cells per well in growth medium consisting of alpha-minimum essential medium (α-MEM; Gibco, USA) supplemented with 10% fetal bovine serum (FBS; Gibco, USA), 1% penicillin-streptomycin (P/S; Gibco, USA). After 24 h, the medium was replaced according to experimental groups: (1) Control (No MMC), α-MEM + 10% FBS + 1% PS without crowders; (2) Ficoll 62.5, medium additionally containing Ficoll PM 70 at 37.5 mg mL^−1^ and Ficoll PM 400 at 25 mg mL^−1^ (total 62.5 mg mL^−1^); (3) Ficoll 125, medium containing Ficoll PM 70 at 75 mg mL^−1^ and Ficoll PM 400 at 50 mg mL^−1^ (total 125 mg mL^−1^); (4) PEG 62.5, medium supplemented with PEG (Mᵣ ≈ 8000) at 62.5 mg mL^−1^; and (5) PEG 125, medium supplemented with PEG (Mᵣ ≈ 8000) at 125 mg mL^−1^. Media were changed every 2–3 days with maintenance of the respective crowder concentrations. Cell viability under different MMC conditions was assessed on Days 1 and 7 viability by staining with calcein-AM (Invitrogen, USA) and ethidium homodimer-1 (Invitrogen, USA), followed by fluorescent imaging using a confocal microscope (TCS SP8 X, Leica, Germany). Cell metabolic activity was evaluated using the CCK-8 assay (Chromo-CK, Monobio, Republic of Korea) on Days 1, 4, and 7 according to the manufacturer’s instructions. Extracellular matrix production was assessed by quantifying collagen deposition using the Sircol™ Soluble Collagen Assay (Biocolor Ltd.) on Day 7 following the manufacturer’s protocol.

#### 2.1.2 ECM harvesting

The mouse calvaria-derived pre-osteoblast cell line MC3T3-E1 (passage 4–14, American Type Culture Collection, USA) was cultured in 150-mm cell culture dishes using alpha-minimum essential medium (α-MEM; Gibco, USA) supplemented with 10% fetal bovine serum (FBS; Gibco, USA), 1% penicillin-streptomycin (P/S; Gibco, USA), 50 μg mL^−1^ L-ascorbic acid 2-phosphate sesquimagnesium salt hydrate (AA2P; Tokyo Chemical Industry Co., Japan), and 10 mM β-glycerophosphate disodium salt pentahydrate (β-GP; EMD Millipore, USA). Two culture conditions were employed: conventional culture (control) and macromolecular crowding (MMC) culture. The conventional culture group refers to standard MC3T3-E1 culture conditions without the addition of crowding agents, whereas the MMC group was cultured in the same basal medium supplemented with macromolecular crowding agents. For the MMC group, additional crowding agents, Ficoll 400 (25 mg mL^−1^) and Ficoll 70 (37.5 mg mL^−1^) (Sigma-Aldrich, USA), were added to the culture medium. Cells were maintained at 37 °C in a humidified atmosphere of 5% CO_2_, with medium replenished every 2–3 d. After 4 weeks of culture, cells were decellularized using one of the following protocols: (1) 0.5% (v/v) sodium dodecyl sulfate (SDS), (2) 0.5% (v/v) Triton X-100, or (3) 100 mM ammonium hydroxide (NH_4_OH) at 37 °C for 2 h, followed by DNase I treatment (50 U mL^−1^, 37 °C, 30 min) to ensure complete removal of cellular material. Non-decellularized samples were designated as the native group. The dECM was collected using a cell lifter, rinsed three times with phosphate-buffered saline (PBS), and rinsed once with sterile water. The harvested ECM was freeze-dried, and the lyophilized ECM was subsequently used for dry weight measurement, DNA, GAG, and collagen quantification assays.

#### 2.1.3 Histological analysis

To evaluate ECM composition, MC3T3-E1 cells were seeded at a density of 1 × 10^4^ cells/well in 24-well plates and cultured for 4 weeks with or without MMC treatment. Decellularized samples were prepared using the NH_4_OH + DNase I protocol detailed in Section 2.1.1. Samples were fixed with 4% paraformaldehyde and permeabilized using 0.2% (v/v) Triton X-100 for 10 min. For GAG staining, the fixed samples were immersed in 3% Alcian blue solution (Sigma-Aldrich, USA) for 1 min, followed by nuclear fast red staining for 30 min. For collagen I visualization, samples were stained with picrosirius red solution (VitroVivo Biotech, USA) for 1 h, according to the manufacturer’s protocol. After staining, samples were dehydrated and imaged using an optical microscope.

#### 2.1.4 Immunofluorescence analysis

Immunofluorescence analysis was performed to evaluate ECM components and confirm the efficacy of the decellularization protocol. MC3T3-E1 cells were seeded at a density of 1 × 10^4^ cells/well in 24-well plates and cultured for 4 weeks with or without MMC treatment. Decellularized samples were prepared using the NH_4_OH + DNase I protocol detailed in Section 2.1.1. Fixed samples were permeabilized with 0.2% (v/v) Triton X-100 for 10 min and blocked with 1% (w/v) bovine serum albumin (BSA) for 1 h. Samples were stained with 4ʹ,6-diamidino-2-phenylindole (DAPI) (Sigma-Aldrich, USA) to visualize cell nuclei. To evaluate specific ECM components, samples were incubated overnight at 4 °C with primary antibodies against collagen types I (ab270993), collagen types III (ab184993), and collagen types V (ab7046), biglycan (BGN) (ab58562), and osteonectin (ON) (ab290636) (1:100 dilution, Abcam, UK). After washing, samples were incubated for 1 h with Alexa Fluor 488-conjugated secondary antibodies (1:200 dilution, Thermo Fisher Scientific, USA) and counterstained with DAPI (1:500 dilution). Fluorescent images were acquired using a confocal microscope (TCS SP8 X, Leica, Germany).

### 2.2 Preparation of CD-ECM bioink

MC3T3-E1 cells (passages 4–14, American Type Culture Collection, USA) were cultured in 150-mm cell culture dishes in either MMC-supplemented medium at an initial density of 1 × 10^6^ cells/dish. After 4 weeks, cells were decellularized using 100 mM NH_4_OH at 37 °C for 2 h, followed by DNase I treatment (50 U mL^−1^, 37 °C, 30 min). The dECM was collected using a cell lifter, rinsed three times with PBS, and rinsed once with sterile water.

The harvested ECM was freeze-dried and weighed prior to digestion. To prepare CD-ECM solutions at defined concentrations (e.g., 2% w/v), the appropriate mass of lyophilized CD-ECM (e.g., 200 mg for a final volume of 10 mL) was solubilized in pepsin (1 mg mL^−1^ in 0.5 M acetic acid). The mixture was stirred at 500 rpm for approximately 48 h at 25 °C until complete solubilization was achieved. Following digestion, the solution was neutralized to physiological pH (7.2–7.4) by dropwise addition of NaOH under cold conditions, and sterile distilled water was added as needed to reach the final volume (10 ml). The neutralized CD-ECM was maintained in a soluble pre-gel state at 4 °C prior to printing. Thermal gelation behavior was verified separately by incubating aliquots at 37 °C, where the solution underwent temperature-induced gelation. To ensure reproducibility and assess batch-to-batch consistency, all functional stability assessments and biological evaluations were performed using bioinks prepared from at least three independent batches of harvested CD-ECM.

### 2.3 CD-ECM characterization

#### 2.3.1 DNA, GAG, and collagen quantification

The freeze-dried ECM was digested and purified using the GeneJET Genomic DNA Purification Kit (Thermo Fisher Scientific, USA), following the manufacturer’s instructions. DNA concentration in the resulting lysate was measured using a PicoGreen assay kit (Thermo Fisher Scientific, USA). Sulfated GAGs were extracted using a papain digestion protocol following the manufacturer’s instructions of the Blyscan™ Sulfated Glycosaminoglycan Assay (Biocolor Ltd.), including incubation at 65 °C for overnight in papain extraction buffer, followed by centrifugation to collect the supernatant for analysis. Similarly, collagen was extracted and quantified according to the manufacturer’s protocol of the Sircol™ Soluble Collagen Assay (Biocolor Ltd.), including pepsin-based solubilization under acidic conditions (1 mg mL^−1^ in 0.5 M acetic acid) followed by centrifugation to collect the supernatant for analysis prior to dye-binding quantification.

#### 2.3.2 Rheological analysis

Rheological properties of neutralized collagen and CD-ECM solutions prepared as described above were analyzed using a rheometer (DHR-1, TA Instruments, USA). Viscosity was measured at 4 °C across a shear rate range of 0.01–1000 s^−1^. To assess thermal response, an oscillation temperature ramp test was performed by heating the samples from 4 °C to 37 °C at a rate of 5 °C min^−1^ for 50 min. Viscoelastic behavior was evaluated using an oscillation frequency sweep test conducted for 300 s at 2% strain, covering frequencies from 0.1 to 100 Hz at 37 °C. These rheological tests provided critical insights into the mechanical and flow properties of ECM solutions, which are essential for evaluating their suitability as bioinks.

### 2.4 3D bioprinting of CD-ECM bioink

Extrusion-based 3D bioprinting was performed using an in-house-developed bioprinting system. A sacrificial rheology modifier of 2 wt% Carbopol (CBP) solution was prepared by dissolving the CBP powder in distilled water under magnetic stirring for 30 min, neutralized with NaOH to pH 7, homogenized using a planetary centrifugal mixer (ARE-310, THINKY, USA), and sterilized under UV light for 2 h. A 2 wt% MMC-CD-ECM solution containing 1 × 10^7^ cells mL^−1^ was prepared, followed by the addition of riboflavin (RF; 300 μM) and sodium persulfate (SPS; 10 mM) as photoinitiators. For composite bioink, 0.25 wt% α-TCP was incorporated into the CD-ECM solution before adding cells and photoinitiators, based on previously established osteogenic conditions [19,20]. The CD-ECM or CD-ECM/α-TCP solution was then mixed with CBP at a 2:1 vol ratio (CBP:CD-ECM) to obtain a homogeneous printable bioink. The printing conditions were as follows: syringe temperature, 18 °C; bed temperature, 25 °C; pressure, 60 kPa; speed, 100 mm s^−1^; nozzle diameter, 280 μm. The printed constructs were photo-crosslinked under 405 nm light for 30 s, washed in PBS, and immersed in cell medium to remove sacrificial CBP. Printability was quantitatively evaluated using the coefficient of variation (CV) of filament diameter and the filament spread ratio (FSR). Filament diameters were measured from optical images of printed filaments. The CV was calculated as the ratio of the standard deviation to the mean filament diameter, expressed as a percentage, and was used to assess filament uniformity. Lower CV values indicate more consistent filament deposition. The FSR was calculated as the ratio of the measured filament diameter to the nozzle diameter, reflecting the extent of filament spreading after extrusion. An FSR value close to 1 indicates minimal spreading and optimal print fidelity.

Light-assisted 3D bioprinting was performed using a custom-designed micro-projection stereolithography system (LITHO MICRO, Plancklab Inc., South Korea) equipped with a 405 nm near-UV LED light source and a digital micromirror device (DMD; pixel pitch, 35 μm). A PDMS-coated polystyrene Petri dish served as the bioink vat. Approximately 2 mL of bioink was loaded into the vat, and the ambient temperature during printing was maintained at 4 °C. Printing parameters were as follows: layer thickness, 100 μm; exposure time, 8 s/layer; lifting distance, 5 mm; and lifting speed, 2 mm s^−1^. After printing, the constructs were gently rinsed with cold PBS to remove uncrosslinked residues. The resulting constructs were then analyzed using Fourier transform infrared (FTIR) spectroscopy (FT/IR-4200, JASCO, USA) to confirm the removal of Carbopol and the preservation of CD-ECM. FTIR spectra were collected over a wavenumber range of 400–4000 cm^−1^ at a spectral resolution of 4 cm^−1^.

### 2.5 In vitro evaluation of CD-ECM-based hydrogels and bioprinted constructs

#### 2.5.1 Preparation and cell culture

MC3T3-E1 Subclone 4–6 cells (American Type Culture Collection, USA) were encapsulated within a 1 wt% CD-ECM hydrogel at a density of 1 × 10^7^ cells mL^−1^ to generate cell-laden hydrogel constructs. The same cell density (1 × 10^7^ cells mL^−1^) was used for CD-ECM bioprinted samples. For CD-ECM/α-TCP bioprinted constructs, human bone marrow-derived mesenchymal stem cells Subclone 2-3 (hBMMSCs, CEFOBIO, Seoul, South Korea) were employed. Samples were incubated at 37 °C in a humidified atmosphere containing 5% CO_2_ under standard culture conditions. To sustain cell viability and proliferation, the culture medium (α-MEM; Gibco, USA) supplemented with 10% FBS and 1% P/S was replenished every 2–3 d. Beginning on Day 4, an osteogenic medium containing 50 μg mL^−1^ AA2P (Tokyo Chemical Industry Co., Japan) and 10 mM β-GP (EMD Millipore, USA) was introduced to promote osteogenic differentiation. Samples were maintained under these conditions for defined periods prior to analyses.

#### 2.5.2 Cell cytotoxicity, proliferation, and viability

Cell cytotoxicity was assessed using a cell viability assay kit (Chromo-CK, Monobio, Republic of Korea). Samples were immersed in culture medium containing CCK-8 solution (10:1) at 37 °C for 2 h. Subsequently, 200 μL of the resulting solution was transferred to a transparent 96-well plate, and absorbance was measured at 450 nm using a spectrophotometer (SpectraMax M2; Molecular Devices, USA). Cell proliferation and viability were visualized by staining encapsulated cells with calcein-AM (Invitrogen, USA) and ethidium homodimer-1 (Invitrogen, USA). Fluorescent images were captured using a confocal microscope (TCS SP8 X, Leica, Germany). Cell proliferation was quantified by measuring total DNA content using a PicoGreen assay (Thermo Fisher Scientific, USA) after lysis in CellLytic M buffer (Sigma-Aldrich, USA).

#### 2.5.3 Osteogenic activities

Osteogenic differentiation was evaluated after 3 weeks of cell culture. Calcium content after 14 days of culture, quantified using a colorimetric o-cresolphthalein complexone (CPC) assay (Calcium Reagent Set, Pointe Scientific, Belgium) according to the manufacturer’s instructions, where calcium reacts with CPC in alkaline conditions to form a purple complex measurable by absorbance (at 570 nm). Before analysis, hydrogel samples containing encapsulated cells were lysed with CellLytic M buffer (Sigma-Aldrich, USA), and the resulting lysate was processed following the manufacturer’s instructions. Subsequently, 200 μL of the reaction solution was transferred to a clear 96-well plate, and absorbance was measured at 570 nm using a spectrophotometer. Alkaline phosphatase (ALP) activity was determined using p-nitrophenyl phosphate (pNPP) as a substrate, with absorbance measured at 405 nm.

Gene expression of osteogenic markers was analyzed using quantitative real-time polymerase chain reaction (qPCR) and reverse transcription polymerase chain reaction (RT-PCR). RNA was extracted using RNAiso reagent (Takara, Japan), reverse-transcribed into complementary DNA (cDNA), and amplified using a thermal cycler (C1000; Bio-Rad, USA) with the following program: 5 min at 25 °C, 1 h at 45 °C, and 5 min at 95 °C. Gene expression was quantified using TB Green Fast qPCR Mix (Takara, Japan) and a real-time PCR system (TP900, Takara, Japan). Relative expression was normalized using the 2^−ΔΔCT^ method with glyceraldehyde-3-phosphate dehydrogenase (GAPDH) as the reference gene. Samples cultured for 1 week were analyzed for ALP, runt-related transcription factor 2 (RUNX2), and collagen type I alpha 1 (COL1A1) expression, while samples cultured for 3 weeks were analyzed for osteocalcin (OCN) and osteopontin (OPN) expression. The primer sequences used for qPCR analysis are listed in Table 1.

**Table 1:** Primer sequences used for quantitative real-time PCR (qPCR) analysis of osteogenic gene expression in MC3T3-E1 pre-osteoblasts and human bone marrow-derived mesenchymal stem cells (hBMMSCs).

Cell Type	Gene	Forward Primer (5′–3′)	Reverse Primer (5′–3′)
MC3T3-E1	RUNX2	TGGTTACTGTCATGGCGGTA	TCTCAGATCGTTGAACCTTGCTA
	COL1A1	GGCAACAGGAAGGCCAGTA	AGGTGATTGGTGGGATGTCTTC
	ALP	ATGGGAGGGGTGCTCTCACA	CCATGATGACCTGATGTCC
	OCN	CACTCCTCGCCTATTGGC	CCCTCCTGCTTGGACACAAAG
	OPN	TGACCAGTTCGGTGCTGTG	CTTGGAAGGGTGTGACTTGT
	GAPDH	AGGTCGGTGTGAACGGATTTG	TGTAGACCATGTAGTTGAGGTCA
hBMMSC	RUNX2	GCCAATCCCTAAGTGTGGCT	ACATAGGTCCCCATCTGCCT
	COL1A1	CCAGCCGCAAAGAGTCTACA	CTTGGGTCCCTCGACTCCTA
	ALP	CAGGCCGCCTTCATAAGCA	GTGCCGATGGCCAGTACTAA
	OCN	GTTTGGCTTTAGGGCAGCAC	GGGCAGCACAGGTCCTAAAT
	OPN	CCTTGCTTGGGTTTGCAGTC	TTCTGTGGCGCAAGGAGATT
	GAPDH	CCATCCAATCGGTAGTAGCG	GTAACCCGTTGAACCCCATT

#### 2.5.4 Immunofluorescence analysis

Samples collected at Week 4 were fixed with 4% paraformaldehyde for 30 min, permeabilized with 0.2% (v/v) Triton X-100 for 10 min, and blocked with 1% (w/v) BSA (Sigma-Aldrich, USA) for 1 h. Subsequently, the samples were incubated overnight at 4 °C with anti-OCN antibody (rabbit polyclonal antibody, ab93876, 1:200 dilution, Abcam, UK) to evaluate osteogenic differentiation. The samples were then stained for 1 h with phalloidin (1:200 dilution), DAPI (1:500 dilution), and Alexa Fluor 594-conjugated secondary antibody (1:200 dilution; Thermo Fisher Scientific). The stained samples were visualized using a confocal microscope (TCS SP8 X, Leica, Germany). Fluorescence intensity was analyzed using ImageJ (NIH, USA) by calculating the mean gray value within defined regions of interest (ROIs) to obtain relative fluorescence intensity.

### 2.6 Statistical analysis

Statistical analysis was conducted using one-way analysis of variance (ANOVA) followed by Tukey’s post-hoc test to determine significant differences between groups. All analyses were performed using Origin software (OriginLab Corporation, USA). Each experiment was conducted in triplicate, with four samples per group. A *p*-value <0.05 was considered statistically significant.

## 3 Results and discussion

### 3.1 Optimization of macromolecular crowding conditions

To determine the optimal macromolecular crowding (MMC) condition for subsequent experiments, cells were cultured in the absence of MMC (conventional culture) or in MMC media containing different crowders and concentrations. Two commonly used synthetic crowders, Ficoll and Polyethylene glycol (PEG), were evaluated at total concentrations of 62.5 mg mL^−1^ and 125 mg mL^−1^. Cell viability was first assessed using live/dead staining (Fig. S1a). At Day 1, most MMC conditions maintained high cell viability, indicating general cytocompatibility. However, increased cell death was observed in the PEG 125 mg mL^−1^ group compared with other conditions. This observation was consistent with cell metabolic activity measured using the CCK-8 assay (Fig. S1b), where PEG 125 mg mL^−1^ exhibited the lowest metabolic activity among all conditions.

By Day 7, the differences between conditions became more pronounced. The PEG 125 mg mL^−1^ group showed a marked increase in cell death and reduced metabolic activity, whereas cells cultured under the Ficoll 62.5 mg mL^−1^ condition maintained high viability and the highest metabolic activity. The PEG 62.5 mg mL^−1^ and Ficoll 125 mg mL^−1^ groups displayed intermediate performance. Extracellular matrix production was further evaluated by quantifying collagen deposition (Fig. S1c). All MMC-treated groups, except PEG 125 mg mL^−1^, enhanced collagen deposition compared with the conventional culture condition. Notably, the Ficoll 62.5 mg mL^−1^ group showed the highest collagen production, approximately two-fold higher than Ficoll 125 mg mL^−1^ and PEG-treated groups.

The effectiveness of macromolecular crowding is better interpreted in terms of fractional volume occupancy (FVO) rather than mass concentration alone. Physiologically relevant crowding conditions have been estimated to correspond to an FVO of approximately 10–20%, reflecting the highly crowded macromolecular environments present in biological systems [21]. The Ficoll 70/400 mixture used in this study (37.5 mg mL^−1^ and 25 mg mL^−1^, respectively) corresponds to an estimated FVO of ∼17%, which falls within this range (Table S1). This moderate crowding condition is expected to promote extracellular matrix assembly by increasing the effective concentration of secreted proteins through excluded-volume effects.

In addition, the use of a polydisperse crowder system composed of Ficoll molecules with different molecular sizes has been reported to generate more efficient volume exclusion than monodisperse systems [21]. Such mixed crowder environments improve macromolecular packing and facilitate collagen fibrillogenesis, while avoiding excessive increases in medium viscosity or osmotic stress [21]. Consequently, Ficoll 70/400 cocktails at total concentrations of approximately 60–70 mg mL^−1^ have been widely adopted in macromolecular crowding studies to enhance extracellular matrix deposition in vitro [14,[21], [22], [23], [24]]. This concentration range provides an effective crowding environment that accelerates ECM assembly while maintaining cytocompatibility and adequate nutrient diffusion. By contrast, higher crowder concentrations substantially increase the fractional volume occupancy of the culture medium. In our screening experiments, both Ficoll 125 mg mL^−1^ and PEG-based MMC conditions corresponded to markedly higher FVO values. Such highly crowded environments are likely to increase medium viscosity and restrict nutrient and oxygen diffusion, thereby impairing cell viability and ECM secretion [21,22,25]. Consistent with this interpretation, these high-FVO conditions showed reduced metabolic activity and lower collagen deposition compared with the Ficoll 62.5 mg mL^−1^ condition.

Taken together, these observations indicate that Ficoll 62.5 mg mL^−1^ represents an optimal crowding window for CD-ECM production in our system, providing enhanced ECM deposition while preserving high cell viability.

### 3.2 CD-ECM decellularization and characterization

**Fig. 2:**
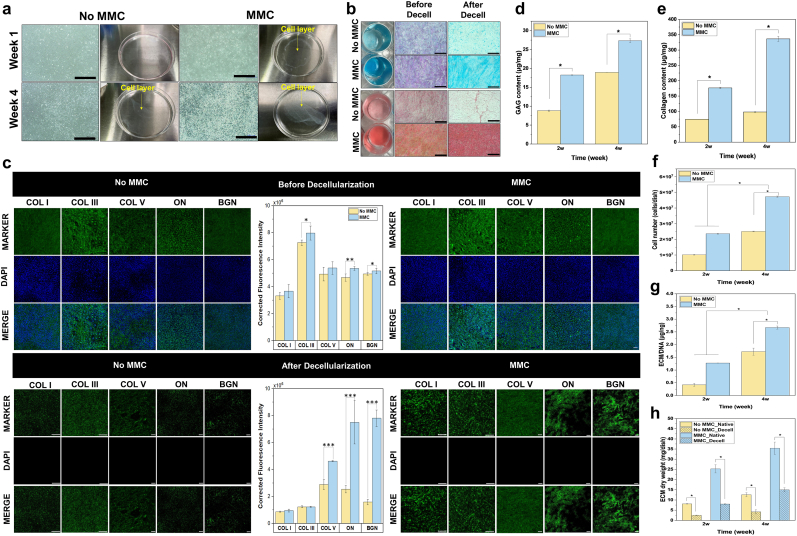
Fabrication, decellularization, and characterization of CD-ECM with and without macromolecular crowding (MMC) treatment. (a) Microscopic and macroscopic images of extracellular matrix (ECM) deposition after one and 4 weeks of culture, showing enhanced ECM formation under MMC conditions. Scale bar = 200 μm. (b) Histological staining using Alcian blue (GAGs, blue) with Nuclear Fast Red counterstaining (nuclei, red), and Picrosirius Red staining (collagen, red) performed separately, before and after decellularization. The staining shows effective cell removal while preserving matrix components, with more pronounced retention in MMC-treated samples. Scale bar = 100 μm. (c) Immunofluorescence analysis and quantitative assessment of ECM components, including collagen type I (COL I), collagen type III (COL III), collagen type V (COL V), osteonectin (ON), and biglycan (BGN), before and after decellularization. Corrected total cell fluorescence (CTCF) analysis demonstrated significantly higher retention of ECM proteins and GAGs in MMC-treated samples compared to controls. Scale bar = 100 μm. (d, e) Biochemical quantification of GAG and collagen content, showing significant enhancement in MMC-treated samples. (f–h) Quantification of ECM yield: (f) cell number, (g) ECM/DNA ratio, and (h) ECM dry weight, demonstrating improved ECM production under MMC conditions. Data are presented as mean ± SD (n = 4 per group). Statistical significance: *∗p* < 0.05, ∗*∗p* < 0.01, *∗∗p* < 0.001. (For interpretation of the references to color in this figure legend, the reader is referred to the Web version of this article.)

During Week 1 of culture, under MMC conditions, the cell layer exhibited a translucent, white matrix-like appearance on the culture surface, which became macroscopically visible (Fig. 2a). In contrast, this appearance was less apparent in the conventional culture group (without MMC) (Fig. 2a). By Week 4, the cell layer appeared more opaque and visually pronounced in the MMC-treated group compared to the control. Microscopic observations revealed densely packed cells with well-preserved morphology in the MMC group.

Following optimization of the decellularization protocols, ammonium hydroxide (NH_4_OH) was selected as the optimal method due to its effective removal of cellular components while preserving ECM integrity, whereas SDS and Triton X-100 were excluded (Fig. S2). Using the NH_4_OH-based protocol, we compared ECM preservation between MMC-treated and control groups. Histological and immunofluorescence analyses confirmed effective decellularization and retention of key ECM components (Fig. 2b and c). Alcian Blue staining revealed GAG-rich regions (blue), and nuclei were counterstained with Nuclear Fast Red (red). In pre-decellularization samples, the apparent purple coloration arises from the overlap of the red nuclear stain and the surrounding blue matrix. This was more evident in the MMC-treated group, where densely distributed cells with preserved morphology were observed (Fig. 2b). After decellularization, nuclear material was completely removed, as evidenced by the absence of purple staining, confirming successful cellular clearance without compromising the ECM. Notably, the blue-stained GAGs, which exhibited a more fiber-like structure, were better preserved in the MMC-treated group, suggesting that MMC facilitated the formation of a denser and more stable ECM network during matrix assembly. The enhanced preservation of GAGs is especially significant, as these molecules are critical for maintaining tissue mechanical properties—including elasticity and tensile strength—and play an essential role in supporting ECM functionality during tissue regeneration [[26], [27], [28]].

Sirius red staining for collagen further demonstrated clear differences between MMC-treated and control groups (Fig. 2b). The intensity of red staining was markedly higher in the MMC-treated group, indicating that MMC treatment effectively preserved the collagen matrix during decellularization. Collagen retention is crucial for tissue engineering applications, as it provides mechanical strength, maintains tissue integrity, and supports cellular activities such as migration, proliferation, and differentiation [29,30]. In the context of 3D bioprinting, collagen within the bioink contributes to photo- or chemical crosslinking, enhancing the mechanical stiffness and structural fidelity of printed constructs, which is essential for maintaining shape accuracy and supporting subsequent cell activities [31,32].

Immunofluorescence analysis of bone-specific markers—including collagen types I, III, and V, ON, and BGN—was performed to evaluate the efficacy of the MMC treatment on ECM deposition and preservation (Fig. 2c). Before decellularization, the MMC-treated group exhibited markedly higher fluorescence intensities across all bone-specific markers compared to the control, indicating that MMC promotes enhanced ECM deposition during culture. After decellularization, DAPI staining confirmed the complete removal of nuclei, while the intensity of green fluorescence for bone markers remained significantly higher in the MMC-treated group, demonstrating superior preservation of ECM components. Quantitative fluorescence analysis based on corrected total cell fluorescence (CTCF) further confirmed that MMC-treated samples retained higher levels of collagen types I, III, and V, ON, and BGN than controls. These markers play complementary roles in maintaining ECM functionality and supporting osteogenesis: collagen types I and III provide tensile strength, collagen type V regulates fibril assembly, ON facilitates collagen binding and mineralization, and BGN enhances osteogenic differentiation by activating the TGF-β, ERK/Runx2, and Smad signaling pathways, promoting ALP activity, osteogenic gene expression, and mineralization, while its GAG chains facilitate BMP-4-mediated osteoblast differentiation [[33], [34], [35], [36]]. Collectively, these findings demonstrate that MMC not only enhances ECM deposition during culture but also preserves bioactive components after decellularization, thereby creating a more favorable microenvironment for osteogenic differentiation, which is essential for bone tissue engineering applications.

To further validate these observations, biochemical assays were performed to quantify GAG and collagen levels in the ECM (Fig. 2d and e). By Week 2, both components were already higher in the MMC-treated group compared to the control, and these differences became more pronounced by Week 4. GAG content showed a steady, time-dependent accumulation, with the MMC-treated group reaching nearly double the levels of the non-MMC group at Week 4 (Fig. 2d). Similarly, collagen was significantly enriched in the MMC-treated samples, confirming that MMC promotes both ECM deposition and retention of key biological components (Fig. 2e). Histological staining, immunofluorescence analysis, and biochemical assays collectively indicated that MMC treatment enhanced overall ECM deposition compared with the control group. However, because these techniques assess collagen using different detection principles, the magnitude of increase observed by each method may not appear identical. Picrosirius Red staining reflects total fibrillar collagen accumulation by binding to the triple-helical structure of collagen fibers, whereas immunofluorescence analysis detects specific collagen isoforms (e.g., types I, III, and V) through antibody-based epitope recognition. Consistent with these observations, quantitative collagen analysis (Fig. 2e) also confirmed a significant increase in collagen content under MMC conditions.

Batch-to-batch analysis revealed that GAG and collagen contents remained highly consistent across independent ECM preparations, regardless of decellularization (Fig. S3a). This high reproducibility ensures uniform ECM quality and supports the scalable preparation of CD-ECM bioinks. These findings align with previous reports showing MMC significantly enhances ECM assembly by accelerating collagen type I production and increasing the density of fibrous structures within the ECM [37,38]. MMC facilitates protein–protein interactions and accelerates fibrillogenesis, enhancing collagen type I assembly through its interactions with fibronectin, which serves as a nucleation site for fibril formation [39]. Additionally, MMC increases fibronectin deposition while inhibiting sulphatase activity in endothelial cells, leading to greater GAG accumulation and overall enrichment of ECM composition and functionality [[40], [41], [42]].

Given that MMC enhanced both ECM deposition and preservation, we next examined its effect on overall ECM yield between MMC-treated and control groups (Fig. 2f–h). Cell numbers increased in both groups, but cell proliferation was consistently higher in the MMC-treated group (Fig. 2f). ECM production normalized to DNA content steadily increased from Week 2 to Week 4, with MMC-treated samples producing significantly more ECM per cell compared to the controls (Fig. 2g). Dry weight measurements further confirmed a time-dependent increase, with the MMC-treated group consistently yielding greater amounts of both native and decellularized ECM (Fig. 2h). In our current setup, CD-ECM was produced using 150 mm culture dishes (growth surface area: 145 cm^2^ per dish). Under MMC conditions, each dish yielded approximately 15 mg of lyophilized CD-ECM after 4 weeks of culture, whereas the control group produced 4.17 mg per dish. When normalized to the culture surface area, this corresponds to 0.103 mg/cm^2^ under MMC conditions compared with 0.029 mg/cm^2^ in conventional culture, representing approximately a 3.6-fold increase in ECM production. Based on this yield, approximately 13-14 MMC-treated 150 mm dishes are sufficient to obtain 200 mg of CD-ECM, which is required to prepare 10 mL of 2% CD-ECM bioink. This amount of bioink is already sufficient to fabricate multiple centimeter-scale constructs (e.g., 8∼9 lattice structures of 10 × 10 × 10 mm^3^, accounting for processing losses). These results indicate that MMC substantially improves ECM production efficiency and supports the practical scalability of CD-ECM bioink preparation at the laboratory scale. Although further scale-up using multilayer culture systems such as cell factories or bioreactor-based platforms will be required for large-scale manufacturing, the present results support the feasibility of MMC as a scalable and sustainable strategy for CD-ECM bioink production.

To assess the broader applicability of MMC, we evaluated ECM production across multiple cell types, including hADSCs, human lung fibroblasts (HLFs), and mouse myoblasts (C2C12), validated by ECM dry weight measurements (Fig. S3b and c). Therefore, based on the defined ECM yield per culture surface area and the use of expandable cell culture systems, the approach provides a controlled and repeatable production process and represents a potentially sustainable alternative to tissue-derived ECM sourcing. ECM production was feasible across all tested cell types, though yields varied by origin: MC3T3-E1 cells produced the highest yield, followed by hADSCs, HLFs, and C2C12. Bright-field imaging confirmed that all cell types maintained typical morphology and spreading prior to decellularization (Fig. S3c, **left**). Lyophilized CD-ECMs from all groups displayed distinct morphologies, consistent with yield differences (Fig. S3c, **middle-left**). Importantly, all CD-ECMs could be solubilized in acidic pepsin solution (Fig. S3c, **middle-right**) and subsequently underwent successful thermal gelation at 37 °C (Fig. S3c, **right**), enabled by their collagen content. These results demonstrate that MMC supports the preparation of diverse CD-ECMs with variable yields but consistent functional properties, establishing their feasibility as tissue-specific bioinks for bioprinting applications.

MMC enhances ECM yield by mimicking the dense extracellular environment found in vivo [17]. By increasing the effective concentration of macromolecules in the culture medium, MMC facilitates the secretion and deposition of ECM components, promoting efficient resource utilization [43]. This effect is evident from the higher cell proliferation rates and increased ECM production observed in the MMC-treated group compared to the control (Fig. 2f–h). The consistent increase in ECM dry weight and ECM-to-DNA ratio highlights MMC’s ability to sustain ECM synthesis, making it a valuable tool for scaling up ECM production in tissue-specific bioink fabrication. Collectively, these findings demonstrate that MMC promotes cell proliferation, enhances ECM deposition, and significantly improves overall ECM yield, while broadening applicability across different cell sources. This establishes MMC as a versatile platform for producing CD-ECM bioinks tailored for scalable and tissue-specific bioprinting applications.

### 3.3 Cytocompatibility and osteogenic differentiation of cells encapsulated in MMC-treated CD-ECM hydrogel

**Fig. 3:**
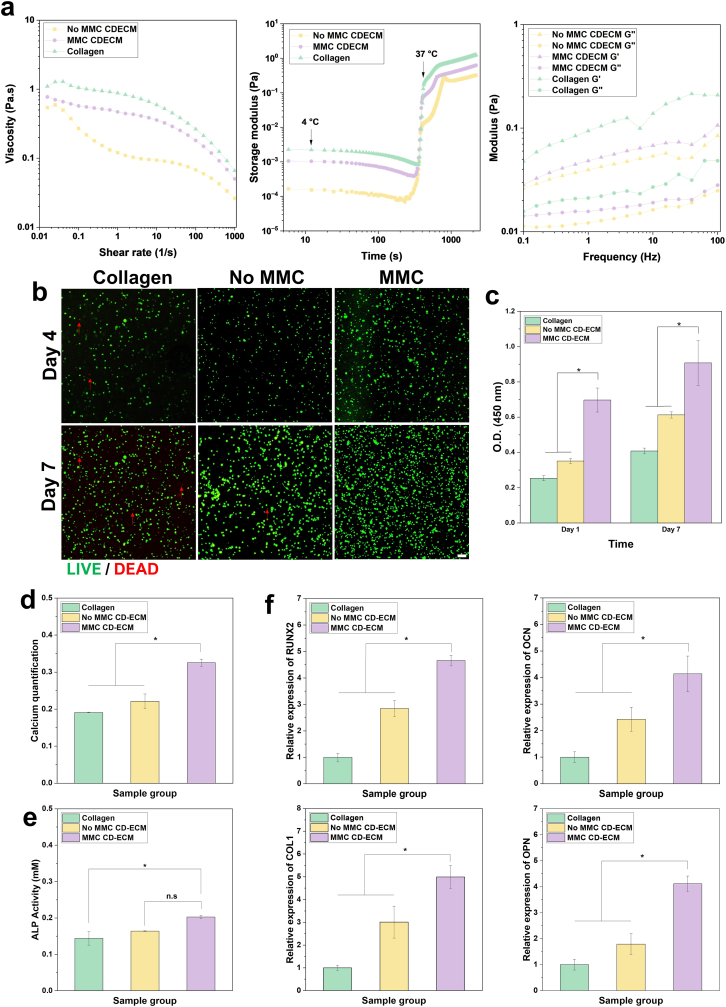
Rheological properties, biocompatibility, and osteogenic potential of CD-ECM bioinks with and without MMC treatment. (a) Rheological properties of porcine collagen (1%), untreated CD-ECM (2%), and MMC-treated CD-ECM (2%). Left: Viscosity profiles at 4 °C showing higher viscosity in MMC-treated CD-ECM, with both bioinks exhibiting shear-thinning behavior advantageous for extrusion-based bioprinting. Middle: Temperature-dependent gelation curves showing liquid-to-gel transition from 4 °C to 37 °C. Right: Frequency sweep analysis showing higher storage (Gʹ) and loss (Gʺ) moduli in MMC-treated CD-ECM, indicating enhanced viscoelastic stability. Both CD-ECM bioinks exhibited lower moduli than porcine collagen. (b) Live/dead staining of encapsulated cells on Days 4 and 7. All bioinks maintained high viability on Day 4. Scale bar = 100 μm. By Day 7, MMC-treated CD-ECM supported markedly higher cell density than untreated CD-ECM and collagen, indicating superior cell survival and proliferation. (c) CCK-8 assay of encapsulated cells on Days 1 and 7. MMC-treated CD-ECM exhibited significantly higher metabolic activity than untreated CD-ECM and collagen hydrogels, suggesting enhanced proliferation. (d) Calcium content after 14 days of culture, quantified using a Calcium Reagent Set (Pointe Scientific, Belgium). MMC-treated CD-ECM showed significantly higher mineral accumulation compared to untreated CD-ECM and collagen, indicating enhanced matrix mineralization. (e) Alkaline phosphatase (ALP) activity after 7 d of culture. MMC-treated CD-ECM exhibited significantly higher ALP activity compared to untreated CD-ECM and collagen, demonstrating enhanced early osteogenic differentiation. (f) Relative expression of osteogenic genes (ALP, COL1A1, RUNX2, OCN, and OPN) after 14 d of culture. MMC-treated CD-ECM significantly upregulated all osteogenic markers compared to untreated CD-ECM and collagen, confirming enhanced osteogenic potential. Scale bars: 100 μm. Data are presented as mean ± SD (n = 4 per group). Statistical significance: ∗*p* < 0.05, ∗∗*p* < 0.01, ∗∗∗*p* < 0.001.

To evaluate the suitability of CD-ECM as a bioink, we first analyzed its rheological properties using porcine collagen (1%, 10 mg mL^−1^) as a reference standard due to its comparable physicochemical characteristics (Fig. 3a, left). CD-ECM bioinks were prepared at 2% (20 mg mL^−1^) to ensure sufficient ECM content and facilitate gel formation. MMC-treated CD-ECM exhibited higher viscosity than untreated CD-ECM across a range of shear rates at 4 °C, while both groups demonstrated pronounced shear-thinning behavior, which is advantageous for extrusion-based bioprinting. Nevertheless, both CD-ECM bioinks showed lower viscosity than porcine collagen.

Temperature-dependent gelation profiles further illustrated the gelation behavior of the bioinks. At 4 °C, CD-ECM from both MMC-treated and non-MMC-treated groups exhibited liquid-like behavior, transitioning sharply to a gel-like state at 37 °C, a characteristic typical of both collagen and CD-ECM (Fig. 3a, middle). Notably, at 37 °C, the storage modulus (Gʹ) consistently exceeded the loss modulus (Gʺ), confirming solid-like behavior under physiological conditions. Frequency sweep analysis revealed that MMC-treated CD-ECM displayed higher G′ and G″ values compared to untreated CD-ECM across all tested frequencies, suggesting improved viscoelastic stability and greater resistance to deformation under dynamic loading (Fig. 3a, right). Nevertheless, both CD-ECM bioinks exhibited lower moduli compared to porcine collagen, consistent with findings reported by Patrawalla et al. [44]. This difference is likely attributable to the nature of 2D cell culture, which typically form less densely organized fibrillar networks with limited intermolecular crosslinking. In contrast collagen derived from native porcine tissue possesses highly aligned and hierarchically organized fibrillar architectures, resulting in greater mechanical stiffness.

Collectively, these findings highlight that MMC treatment enhances viscosity, gelation kinetics, and viscoelastic stability of CD-ECM hydrogels, underscoring their potential as bioinks for extrusion-based 3D bioprinting. MMC-induced molecular compaction likely reduces conformational freedom, resulting in denser and more mechanically robust ECM networks. By stabilizing fibronectin fibers and enhancing cross-linking, MMC strengthens the ECM network and improves structural integrity [39,45]. Nevertheless, MMC-treated CD-ECM exhibited weaker mechanical properties than porcine collagen, which possesses a highly organized fibrillar network and extensive cross-linking developed under native 3D biomechanical stresses [46,47]. Future work should explore 3D culture systems to improve ECM organization and yield, aiming to achieve mechanical properties closer to native tissues.

Next, we examined the biocompatibility of the bioinks by encapsulating MC3T3-E1 pre-osteoblasts in collagen (1%), untreated CD-ECM (2%), or MMC-treated CD-ECM (2%), which were subsequently gelled at 37 °C. Live/dead staining was performed to evaluate cell viability over 7 d (Fig. 3b). On Day 4, all groups exhibited high cell viability, indicated by strong green fluorescence and minimal red signals, confirming cytocompatibility. However, by Day 7, differences in cell density became evident: MMC-treated CD-ECM supported markedly higher cell density compared to untreated CD-ECM and collagen, indicating superior survival and proliferation. Untreated CD-ECM showed moderate cell growth, while the collagen group exhibited relatively sparse cell distribution.

Cell proliferation was further quantified using the CCK-8 assay (Fig. 3c). On both Day 1 and Day 7, the MMC-treated CD-ECM hydrogel exhibited significantly higher metabolic activity compared to the untreated CD-ECM and collagen hydrogels. While the collagen hydrogel provided a baseline for cell compatibility, CD-ECM hydrogels supported greater cellular activity, with MMC treatment amplifying this effect. These results demonstrate that MMC pre-treatment enhances the bioactivity of CD-ECM bioinks by creating a more favorable microenvironment for cell viability and proliferation.

Osteogenic differentiation of encapsulated cells was evaluated by calcium quantification and ALP activity (Fig. 3d and e). Calcium deposition was markedly higher in MMC-treated CD-ECM compared to untreated CD-ECM and collagen, indicating enhanced mineralization (Fig. 3d). Similarly, ALP activity, an early marker of osteoblast differentiation, was significantly elevated in the MMC-treated CD-ECM hydrogel, confirming that MMC pre-treatment promotes both early osteogenesis and matrix mineralization (Fig. 3e). Gene expression analysis (Fig. 3f) further supported these findings. RUNX2, a master regulator of osteoblast differentiation, was significantly upregulated in MMC-treated CD-ECM, while COL1A1, OCN, and OPN—genes associated with ECM production, mineralization, and maturation—were also elevated. Untreated CD-ECM showed moderate improvement over collagen, indicating that the inherent properties of CD-ECM contribute to osteogenic differentiation, while MMC treatment further amplifies these effects.

This enhancement can be attributed to the structural and biochemical characteristics of MMC-treated CD-ECM. During ECM fabrication, MMC promotes the supramolecular assembly and alignment of ECM proteins, resulting in a denser and more organized matrix [14]. Such aligned architectures facilitate improved cell–matrix interactions, supporting actin cytoskeleton organization, enhancing matrix protein secretion, and sustaining cellular metabolic activity [48]. MMC-induced ECM compaction also improves the adsorption and retention of bioactive molecules, creating a more favorable microenvironment for cell adhesion and proliferation [48]. These findings align with previous studies showing that MMC enhances osteogenesis by modulating integrin-mediated signaling and ECM organization. Specifically, MMC upregulates integrins α1 and α2 (ITGA1 and ITGA2), which facilitate collagen-mediated interactions, thereby improving osteogenic differentiation in MSCs [49,50]. Increased ECM accumulation and alignment under MMC further amplify integrin-mediated signaling pathways, enhancing cytoskeletal organization and osteogenic response [51]. Integrin upregulation also influences microtubule assembly, with integrin–microtubule crosstalk regulating cell proliferation, migration, and differentiation [18]. Collectively, these effects highlight the multifaceted role of MMC in promoting osteogenesis, which complements the enhanced osteogenic activity observed in MMC-treated CD-ECM.

### 3.4 Printability and bioprinting performance of CD-ECM bioinks

For the subsequent biofabrication experiments, CD-ECM produced under MMC conditions was exclusively used for bioink preparation. As demonstrated in Fig. 3, MMC treatment significantly increased ECM yield and enhanced osteogenic activity compared with CD-ECM produced under conventional culture conditions. Based on these results, MMC-derived CD-ECM was selected as the primary ECM source for the following bioink formulation and bioprinting experiments. However, despite these biological advantages, CD-ECM bioink inherently exhibit low viscosity, which limits their suitability for extrusion-based 3D bioprinting. To overcome this issue, we incorporated CBP, a rheological modifier, into the CD-ECM formulation to enhance printability. CBP is a crosslinked poly(acrylic acid) microgel that forms a swollen microgel network in aqueous environments [52]. This structure introduces a yield-stress behavior, allowing the bioink to resist flow under low shear conditions while readily flowing under high shear during extrusion. The use of rheological modifiers is a well-established strategy for improving the printability of soft hydrogels, including ECM-derived bioinks. Previous studies have shown that CBP enhances shear-thinning behavior and deposition ability, thereby enabling stable filament formation and improved shape fidelity during extrusion-based bioprinting [53].

**Fig. 4:**
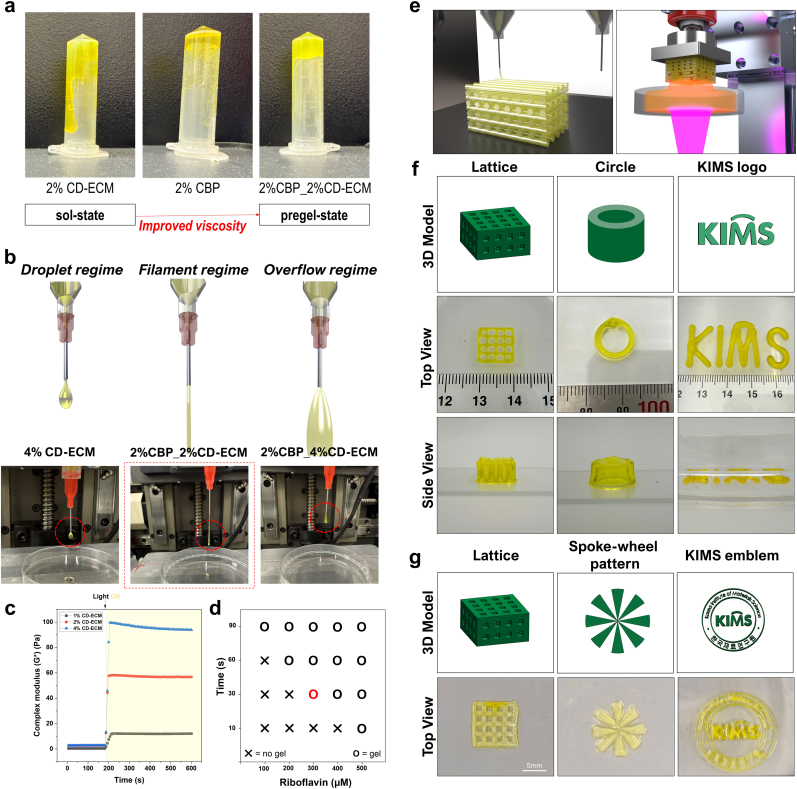
Printability and multi-modal bioprinting performance of CD-ECM bioinks. (a) Inversion test showing the effect of Carbopol (CBP) addition on CD-ECM viscosity. Pure CD-ECM remained in a sol-like liquid state, whereas CBP induced a pre-gel state, confirming improved printability. (b) Extrusion regimes of CD-ECM bioinks, illustrated by schematic and experimental images, demonstrating three distinct behaviors: droplet regime (pure 4% CD-ECM, low viscosity), optimal filament regime (2% CD-ECM + 2% CBP), and overflow regime (4% CD-ECM + 2% CBP, excessive spreading). The 2% CD-ECM +2% CBP formulation produced the most stable and continuous filaments. (c) Photo-crosslinking behavior of CD-ECM bioinks, analyzed via time-sweep rheology. When 365 nm light was applied at 200 s, complex modulus (G∗) increased sharply, confirming rapid sol–gel transition and efficient crosslinking. (d) Optimization of the riboflavin (RF)/sodium persulfate (SPS) photoinitiator system for cytocompatible crosslinking. Phase mapping indicated that 300 μM RF + 10 mM SPS achieved efficient gelation within 30 s while maintaining cytocompatibility. (e) Schematic illustration of the two bioprinting strategies: extrusion-based deposition (left), enabling viscosity-controlled filament printing, and light-assisted digital light processing (DLP) (right), enabling high-resolution photopolymerization. (f) Extrusion-based 3D bioprinting of CD-ECM bioinks, demonstrating accurate fabrication of predefined structures—including a square lattice, circular construct, and custom “KIMS” logo—with high shape fidelity. (g) DLP-based bioprinting of pure CD-ECM bioink, demonstrating successful production of complex geometries—including lattice, spoke-wheel pattern, and the “KIMS” emblem—with smooth surfaces, sharp edges, and excellent architectural precision.

To visually assess the effect of CBP addition, an inversion test was performed (Fig. 4a). Pure CD-ECM remained in a sol-like liquid state when the tube was inverted, whereas CBP inclusion induced a pre-gel-like state, confirming increased viscosity and improved handling. Rheological testing confirmed that adding 2% CBP increased the viscosity of CD-ECM bioinks, with both 2% and 4% CD-ECM formulations exhibiting comparable viscosity profiles when supplemented with CBP (Fig. S4a). The viscosity decreased with increasing shear rate, indicating a pronounced shear-thinning behavior. Such rheological characteristics are advantageous for extrusion-based bioprinting, as they reduce the extrusion force during printing while allowing the bioink to rapidly recover its structure after deposition, thereby supporting filament stability [54].

We next evaluated the extrusion behavior of the prepared formulations to identify the optimal formulation for stable filament formation (Fig. 4b). Pure 4% CD-ECM exhibited droplet-like behavior due to insufficient viscosity, while 4% CD-ECM with 2% CBP showed excessive spreading after extrusion (overflow regime). In contrast, 2% CD-ECM with 2% CBP achieved balanced viscosity, enabling continuous, stable filament extrusion with minimal diameter fluctuation—representing the optimal formulation for extrusion-based bioprinting. Increasing ECM concentration did not necessarily improve printing performance. Although higher ECM concentrations increase viscosity, excessive matrix content may still lead to filament spreading due to insufficient yield stress or slow structural recovery after extrusion. The 2% CD-ECM +2% CBP formulation likely provides a balanced viscoelastic profile, enabling smooth flow through the nozzle while rapidly recovering its structure after deposition, thereby maintaining stable filament geometry [55,56]. Printability was further quantified using two quantitative metrics: the coefficient of variation (CV) for filament diameter and the filament spread ratio (FSR), which compares filament diameter to nozzle diameter (Fig. S4b). The CBP +2% CD-ECM group achieved a CV of 3.53% (well within the acceptable range of CV < 10–15%) [57] and an FSR closest to 1, confirming optimal printing [58].

To further optimize the RF/SPS crosslinking system, gelation efficiency and cytocompatibility were evaluated under varying riboflavin (RF) concentrations and exposure times to identify optimal conditions for collagen/CD-ECM crosslinking while maintaining sufficient mechanical integrity. The RF/SPS system induces crosslinking in collagenous matrices through radical-mediated oxidation of amino acid residues. Under 405 nm visible light, riboflavin is photoexcited and engages in Type I and Type II photochemical reactions, transferring electrons to persulfate, which generates sulfate radicals (SO_4_^•–^) and related reactive species [59]. These radicals oxidize amino acid side chains in ECM proteins, particularly tyrosine, forming tyrosyl radicals that couple to yield covalent dityrosine crosslinks, thereby stabilizing the collagen network. Recent work on photocrosslinkable triple-helical proteins and collagen hydrogels confirms that riboflavin/SPS systems predominantly form di-tyrosine bonds, while additional oxidation of histidine, lysine, and other residues also contributes to intermolecular crosslinking and increased mechanical stiffness without requiring synthetic polymer modification [60].

Time-sweep rheology revealed that light exposure at 200 s induced a sharp increase in complex modulus (G∗), confirming efficient sol–gel transition and crosslinking across all formulations (Fig. 4c). A formulation consisting of 300 μM RF and 10 mM sodium persulfate (SPS) achieved rapid and stable gelation within 30 s of light exposure, providing adequate mechanical robustness for construct handling (Fig. 4d, Fig. S4c). Viability analysis showed no significant differences among RF concentrations on Day 1, indicating that short-term exposure to RF and SPS does not adversely affect cell viability (Fig. S4c). However, by Day 7, cell viability decreased progressively with increasing RF concentration, with a significant reduction observed above 400 μM. In contrast, RF concentrations below 200 μM provided slightly higher long-term viability but required substantially longer gelation times, which can increase overall processing time during bioprinting and potentially induce secondary cell damage due to prolonged handling. Based on these findings, 300 μM RF with 10 mM SPS was identified as the optimal condition for rapid gelation, construct stability, and cytocompatibility for biofabrication applications.

To evaluate the versatility of the developed CD-ECM bioink, we applied it across two bioprinting platforms: extrusion-based deposition and light-assisted digital light processing (DLP) (Fig. 4e). In extrusion-based bioprinting, the bioink successfully fabricated constructs including a square lattice, a circular cylinder, and a custom “KIMS” logo (Fig. 4f). These structures exhibited excellent fidelity, with sharp edges and well-defined geometries at the millimeter scale, confirming that the optimized formulation supports reproducible filament deposition and shape retention.

Following extrusion, the constructs were photo-crosslinked and immersed in culture medium at 37 °C for 24 h to remove CBP. FTIR analysis confirmed the effective removal of CBP after incubation (Fig. S4d). In the printed constructs before washing (“as-printed”), characteristic CBP peaks were observed at ∼1708 cm^−1^ (C=O stretching); however, these peaks disappeared after 24 h. Moreover, clear and well-defined amide I (∼1652 cm^−1^) and amide II (∼1553 cm^−1^) peaks were observed after CBP removal. These peaks closely resemble the spectrum of pure CD-ECM. Importantly, the printed constructs retained their original geometry and mechanical stability after CBP removal, demonstrating that the crosslinked CD-ECM bioinks can retain shape fidelity independently of CBP support.

In parallel, DLP-based bioprinting using pure CD-ECM bioink (without CBP) enabled fabrication of complex 3D architectures, including a lattice, spoke-wheel pattern, and the “KIMS” emblem (Fig. 4g). Light-induced crosslinking produced high-resolution constructs with sharp edges, smooth surfaces, and excellent architectural precision, highlighting the suitability of CD-ECM bioinks for high-resolution photopolymerization.

To the best of our knowledge, only a limited number of studies have explored 3D bioprinting using cell-derived ECM (CD-ECM)–based bioinks. For instance, cornea-specific CD-ECM derived from differentiated hASCs has been incorporated into HA-based formulations to achieve suitable printability, transparency, and cytocompatibility [61]. Similarly, cell sheet–derived ECM bioinks have been developed to enhance biological relevance through naturally accumulated matrix deposition [62]. However, in these approaches, structural stability is primarily achieved through substantial incorporation of polymeric rheological modifiers, resulting in hybrid bioink systems rather than predominantly ECM-based constructs. In addition, pre-crosslinking steps are often required to obtain suitable printability, and the resulting printed structures are typically limited to relatively simple geometries.

In contrast, the present study demonstrates the fabrication of scaffolds predominantly composed of CD-ECM, enabling the generation of complex three-dimensional architectures without reliance on high proportions of secondary structural polymers. By enhancing ECM production prior to processing and employing rapid crosslinking chemistry, CD-ECM was engineered to serve both structural and biological functions, establishing a more matrix-centric and scalable biofabrication strategy. Furthermore, the developed CD-ECM bioink exhibited compatibility with both extrusion-based and DLP bioprinting platforms, allowing the fabrication of structurally precise, tissue-mimetic constructs that maintained stability after CBP removal. These findings suggest that this system represents a more matrix-centric and scalable approach for CD-ECM–based biofabrication.

### 3.5 Osteogenic response of CD-ECM/α-TCP bioprinted constructs

To enhance the osteogenic potential of the MMC-treated CD-ECM system, α-TCP was incorporated into the bioink formulation. Calcium phosphate ceramics are well known for their osteoconductive properties, supporting mineral deposition and providing ionic cues that promote osteogenic differentiation. By combining ECM-derived biochemical signals with the inorganic osteoconductivity of α-TCP, we aimed to create a composite bioink with synergistic effects on cell behavior and bone-like tissue formation.

**Fig. 5:**
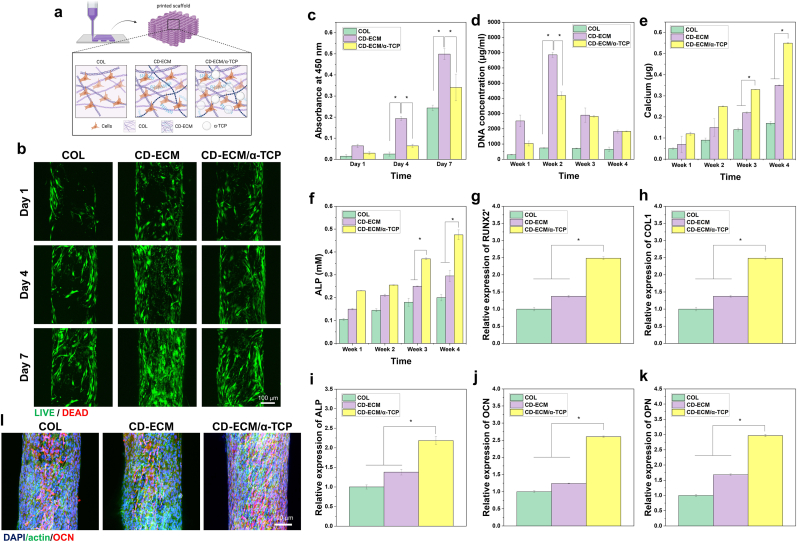
Osteogenic response of CD-ECM/α-TCP bioprinted constructs. (a) Schematic illustration of 3D bioprinted scaffolds composed of collagen (COL), CD-ECM, and CD-ECM/α-TCP composites. (b) Live/dead fluorescence images showing high cell viability of encapsulated MSCs across all scaffolds over 7 d (green = live, red = dead). (c–f) Quantitative analysis of cell proliferation and osteogenic activity: (c) metabolic activity (OD at 450 nm), (d) DNA concentration, (e) calcium deposition, and (f) ALP activity over time, demonstrating enhanced osteogenic responses in the CD-ECM/α-TCP group. (g–k) Gene expression analysis of osteogenic markers (RUNX2, COL1A1, ALP, OCN, and OPN) at Week 4, showing significant upregulation in CD-ECM/α-TCP constructs compared with COL and CD-ECM alone. (l) Maximum intensity projection of merged immunofluorescence staining of actin (green) and osteocalcin (OCN, red) with nuclear counterstain (DAPI, blue) at Week 4, illustrating osteogenic marker expression and cytoskeletal morphology in the bioprinted constructs. Scale bars: 100 μm. Data are presented as mean ± SD (n = 4 per group). Statistical significance: ∗p < 0.05, ∗∗p < 0.01, ∗∗p < 0.001. (For interpretation of the references to color in this figure legend, the reader is referred to the Web version of this article.)

Cytocompatibility, proliferation, and osteogenic differentiation of hBMMSCs were evaluated within 3D bioprinted scaffolds composed of collagen (COL) as control, CD-ECM, and CD-ECM/α-TCP composites (Fig. 5a). All constructs maintained their structure throughout the culture period; however, α-TCP incorporation improved handling stability. The CD-ECM/α-TCP scaffolds maintained their shape during media exchange and routine handling more consistently than the collagen and CD-ECM scaffolds, which occasionally showed minor fragmentation (Fig. S5a). This reinforcement is consistent with the hydrolytic conversion of α-TCP to calcium-deficient hydroxyapatite (CDHA), a dissolution–precipitation process that generates fine CDHA crystals that integrate with the surrounding hydrogel network to enhance mechanical stability [19,20].

Live/dead fluorescence imaging confirmed high cell viability across all groups, with predominantly green fluorescence and minimal red signals at Days 1, 4, and 7 (Fig. 5b). By Day 7, CD-ECM scaffolds displayed a denser and more uniform distribution of live cells, indicating superior cytocompatibility and enhanced cell–matrix interactions compared with collagen and CD-ECM/α-TCP constructs. Quantitative assays (Fig. 5c and d) also showed significant increases in metabolic activity (OD at 450 nm) and DNA content over time in all groups, with CD-ECM scaffolds exhibiting the highest metabolic activity at Day 7 and peak DNA concentration at Week 2. Meanwhile, the increase in DNA content up to 2 weeks in all groups likely reflects an increase in cell number during the early culture phase [19,63]. As the constructs undergo matrix deposition and mineralization, the resulting dense environment may limit available space and the diffusion of nutrients and oxygen [64], which can contribute to a reduction in cell number during prolonged culture [19,65]. Despite the decrease in DNA content, the constructs continued to exhibit strong osteogenic maturation, as evidenced by increased calcium deposition and upregulation of late-stage osteogenic markers (Fig. 5e–j-k).

Osteogenic differentiation was evaluated through biochemical, gene expression, and immunofluorescence analyses to assess MSC maturation within the bioprinted scaffolds. ALP activity and calcium deposition increased progressively throughout the culture period, reaching their highest levels at Week 4 in the CD-ECM/α-TCP group (Fig. 5e and f). Among all scaffolds, CD-ECM/α-TCP exhibited the greatest ALP activity and significantly higher calcium accumulation, confirming superior osteogenic performance. Gene expression analysis further supported these findings, showing marked upregulation of key osteogenic markers, including RUNX2, COL1A1, ALP, OCN, and OPN, in CD-ECM/α-TCP constructs compared with collagen and CD-ECM scaffolds (Fig. 5g–k). Immunofluorescence staining also verified the osteogenic enhancement induced by α-TCP incorporation (Fig. 5l and Fig. S5c). Cells within CD-ECM/α-TCP scaffolds displayed well-organized actin filaments and strong OCN expression along the printed struts, consistent with the quantitative fluorescence analysis showing the highest OCN intensity in this group (Fig. S5b).

The enhanced osteogenic response observed in CD-ECM/α-TCP scaffolds can be attributed to the synergistic interplay between ECM-derived biochemical cues and the osteoconductive properties of α-TCP. The progressive increase in ALP activity and calcium deposition reflects a successful transition from early to late stages of osteogenesis, consistent with effective matrix mineralization. These results align with previous reports showing that calcium phosphate-containing hydrogels provide favorable microenvironments for osteoblast maturation and mineral deposition [19,66,67]. Bioactive ECM components such as collagen and proteoglycans (e.g., BGN) further facilitate osteogenic signaling and reinforce cell–matrix interactions. When combined with α-TCP, which gradually releases calcium and phosphate ions during conversion to CDHA, the composite provides synergistic biochemical and ionic cues that promote osteogenic differentiation. Calcium phosphate-based materials also enhance bone formation by providing surfaces conducive to cell adhesion and spreading and by activating key signaling pathways, including BMP/Smad, TGF-β, and Wnt/β-catenin [68,69].

Collectively, these findings demonstrate that integrating CD-ECM with α-TCP produces a bioactive, osteoinductive matrix that closely mimics the native bone microenvironment, fostering osteogenic signaling, matrix mineralization, and the maturation of functional bone-like tissue for advanced regenerative applications. Importantly, the in vivo biocompatibility and osteoinductive capacity of cell-derived ECM systems have been widely demonstrated, which reported enhanced bone regeneration and osteogenic differentiation when ECM-based scaffolds were implanted in ectopic and orthotopic animal models compared to unmodified controls [70,71]. While the present study establishes the in vitro efficacy of our MMC-enhanced CD-ECM/α-TCP bioink, direct in vivo evaluation of this specific formulation remains necessary. As this work primarily focuses on establishing a scalable CD-ECM bioink production platform, comprehensive in vivo validation will be addressed in future studies to assess tissue integration, remodeling dynamics, and long-term regenerative performance in relevant models.

Despite increasing interest in cell-derived extracellular matrix (CD-ECM) as a biomimetic material, its application as a bioink has remained limited by the low ECM yield typically obtained under conventional culture conditions. In this study, macromolecular crowding significantly enhanced ECM deposition and enabled the establishment of a defined and reproducible CD-ECM production framework. By quantifying production conditions and ECM yield, our results demonstrate that MMC-enhanced CD-ECM can be generated at levels sufficient for practical bioink fabrication, thereby addressing one of the key limitations of conventional CD-ECM systems.

Unlike tissue-derived dECM bioinks that rely on harvested tissues and are often constrained by donor variability and limited scalability, the present approach utilizes culture-expandable CD-ECM and converts MMC-enhanced ECM into a bioink-ready production platform. The resulting CD-ECM bioink demonstrated compatibility with multiple bioprinting modalities, including extrusion-based and DLP-based 3D bioprinting, enabling the fabrication of structurally precise three-dimensional constructs. Furthermore, incorporation of α-tricalcium phosphate (α-TCP) enabled the development of a composite ECM bioink with enhanced osteogenic functionality, illustrating the potential of this system for tissue-specific bioink design.

Notably, MMC-mediated ECM enhancement was also observed across multiple cell types, including C2C12 cells, human fibroblasts, and human adipose-derived stem cells, demonstrating that the platform can be extended to diverse cellular sources for ECM bioink generation. These findings further suggest the feasibility of producing fully human-derived CD-ECM bioinks using human cell sources. Because ECM can be generated directly from patient-derived cells, this strategy may also enable the development of patient-specific ECM microenvironments for personalized regenerative applications.

Together, these results establish a scalable and adaptable CD-ECM biofabrication platform that bridges enhanced ECM production with practical bioink manufacturing for regenerative medicine.

## 4 Conclusion

This study presents a novel strategy for developing a high-yield, biologically functional CD-ECM bioink through MMC. The MMC approach significantly enhanced ECM deposition, preserved key bioactive components (collagen, GAGs, and BGN), and improved the mechanical and rheological properties of the resulting hydrogels. The MMC-enhanced CD-ECM bioink demonstrated excellent printability and was successfully applied in both extrusion-based and DLP bioprinting, enabling the fabrication of structurally precise, cell-laden constructs. In vitro studies confirmed that the bioink provided a cytocompatible microenvironment supporting high cell viability, proliferation, and osteogenic differentiation. Furthermore, the incorporation of α-TCP imparted additional osteoconductive cues, synergistically enhancing osteogenic gene expression, matrix mineralization, and osteoblastic maturation. Overall, MMC-enhanced CD-ECM, particularly when combined with α-TCP, represents a versatile, bioactive, and scalable bioink platform that recapitulates the native bone microenvironment and holds strong potential for advanced 3D bioprinting and bone tissue regeneration applications.

## CRediT authorship contribution statement

**Siwi Setya Utami:** Data curation, Investigation, Methodology, Validation, Visualization, Writing – original draft. **Honghyun Park:** Investigation, Methodology, Validation. **Jueun Kim:** Data curation, Methodology, Validation. **Aram Sung:** Data curation, Investigation, Methodology, Validation. **Min-Ju Choi:** Data curation, Investigation, Methodology, Validation, Visualization. **Ga-Hee Ryoo:** Investigation, Methodology, Visualization. **Chang Woo Gal:** Investigation, Methodology, Validation. **Insup Kim:** Data curation, Investigation, Methodology. **Hui-Suk Yun:** Funding acquisition, Investigation, Supervision, Writing – review & editing. **Yeong-Jin Choi:** Conceptualization, Data curation, Funding acquisition, Investigation, Methodology, Project administration, Supervision, Writing – original draft, Writing – review & editing.

## Declaration of competing interest

The authors declare that they have no known competing financial interests or personal relationships that could have appeared to influence the work reported in this paper.

## Data Availability

Data will be made available on request.
