# Supplementary material for: High-yield cell-derived extracellular matrix bioink via macromolecular crowding for versatile 3D bioprinting
Source: Mater Today Bio. 2026 Apr 17;38:103135. doi: 10.1016/j.mtbio.2026.103135 (PMC13123329; doi:10.1016/j.mtbio.2026.103135)
Supplement: Multimedia component 1 [file mmc1.docx]

**Supporting Information**

**High-Yield Cell-Derived Extracellular Matrix Bioink via Macromolecular Crowding for Versatile 3D Bioprinting**


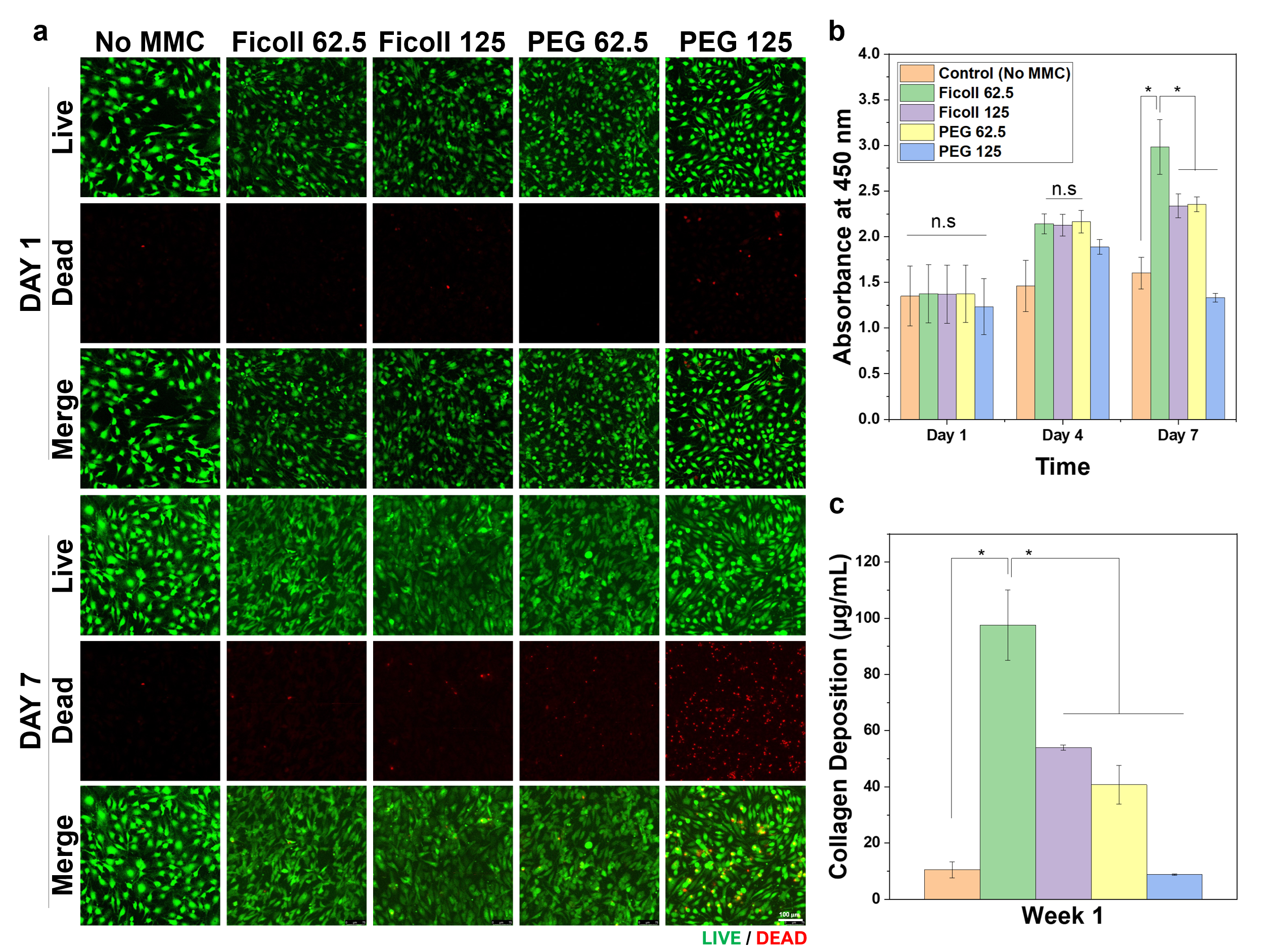


**Supporting Figure S1.** Comparison of macromolecular crowding (MMC) conditions using Ficoll and polyethylene glycol (PEG) at medium (62.5 mg mL⁻¹) and high (125 mg mL⁻¹) concentrations. (a) Live/dead staining of cells cultured under different MMC conditions at Day 1 and Day 7. Ficoll 62.5 mg mL⁻¹ maintained high viability and confluence over time. (b) Cell metabolic activity (CCK‑8) at Day 1, 4, and 7. Ficoll 62.5 mg mL⁻¹ showed a sustained increase in metabolic activity compared with control and other MMC groups. (c) Collagen deposition at Week 1. All MMC conditions increased collagen relative to control, with Ficoll 62.5 mg mL⁻¹ yielding the highest collagen production. Scale bars: 100 μm. Data are mean ± SD; *p < 0.05, n.s. = not significant.

| **Crowder** | **Concentration (mg mL⁻¹ )** | **Molecular weight (Da)** | **R_H_ (nm)** | **FVO (%)** |
| --- | --- | --- | --- | --- |
| Ficoll 70/400 | 37.5 (70) / 25 (400) | 70,000 / 400,000 | 4.0 / 8.0 [1] | 16.7 (8.65 + 8.07) |
| Ficoll 70/400 | 75 (70) / 50 (400) | 70,000 / 400,000 | 4.0 / 8.0 [1] | 33.4 (17.30 + 16.15) |
| PEG 8k | 62.5 | 8,000 | 2.7 [2] | 38.8 |
| PEG 8k | 125 | 8,000 | 2.7 [2] | 77.6 |

**Supporting Table S1.** Summary of macromolecular crowding (MMC) conditions used for fractional volume occupancy (FVO) calculations. FVO values were estimated assuming spherical crowders from their concentration, molecular weight and hydrodynamic radius (RH), and expressed as total FVO with the individual contributions of Ficoll 70 and Ficoll 400 indicated in parentheses where applicable. R_H_ for PEG 8000 was taken from published dynamic light scattering measurements [2].

The fractional volume occupancy (FVO) of each crowder was calculated based on previously published approaches for macromolecular crowding [1, 3]. FVO was calculated assuming spherical crowders as

FVO (%)=$\left\lfloor\frac{4}{3}\pi{R_{H}}^{3}\times\frac{c\times{10}^{-3}\times N_{A}}{M_{W}} \right\rfloor\times100$

where R_H_ is the hydrodynamic radius, c is the crowder concentration (mg mL⁻¹ ), M_w_ is the molecular weight of the crowder (Da), N_A_ is Avogadro’s number. For crowder mixtures (e.g. Ficoll 70 and Ficoll 400), FVO was calculated for each component and summed.

[1] C. Chen, F. Loe, A. Blocki, Y. Peng, M. Raghunath, Applying macromolecular crowding to enhance extracellular matrix deposition and its remodeling in vitro for tissue engineering and cell-based therapies, Adv Drug Deliv Rev 63(4-5) (2011) 277-90.

[2] K. Onuma, N. Furubayashi, F. Shibata, Y. Kobayashi, S. Kaito, Y. Ohnishi, K. Inaka, Relationship between molecular weight of poly(ethylene)glycol and intermolecular interaction of Taka-amylase A monomers, Journal of Crystal Growth 312(9) (2010) 1581-1589.

[3] Q. Liu, H.J. Jiang, Y.D. Wu, J.D. Li, X.H. Sun, C. Xiao, J.Y. Xu, Z.Y. Lin, Carrageenan maintains the contractile phenotype of vascular smooth muscle cells by increasing macromolecular crowding in vitro, Eur J Med Res 29(1) (2024) 249.


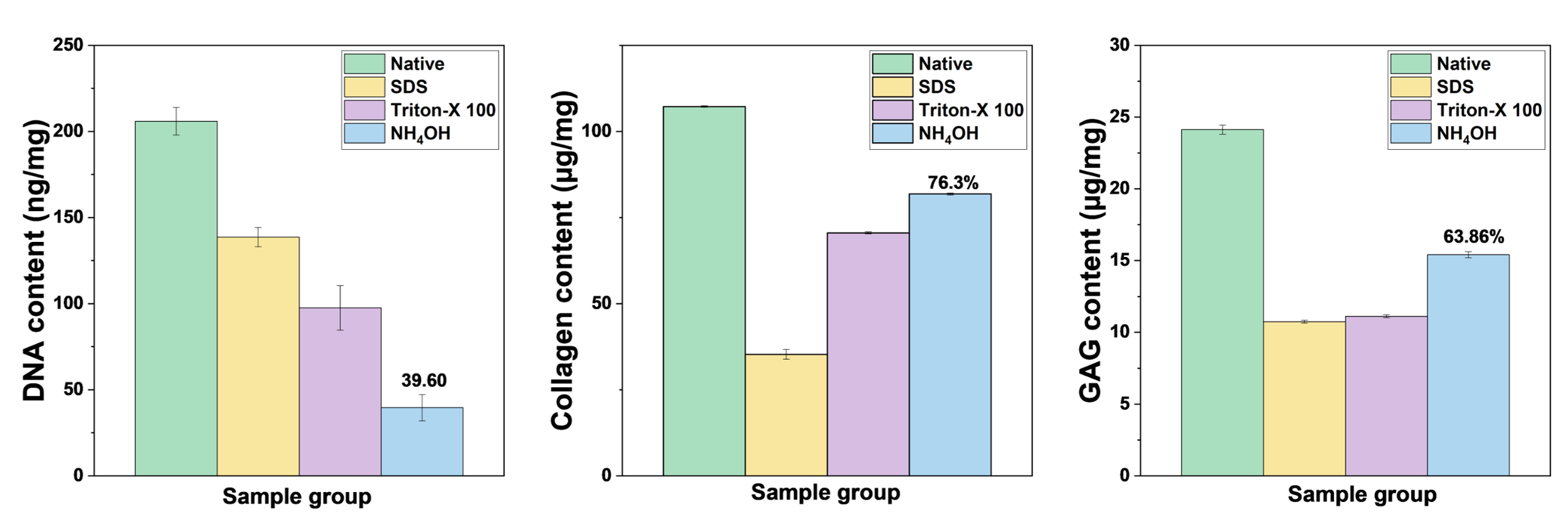


**Supporting Figure S2.** Optimization and evaluation of decellularization protocols. (a) DNA, collagen, and GAG content following decellularization with SDS, Triton X-100, or NH₄OH, highlighting NH₄OH as the most effective method for DNA removal while best preserving collagen (76.3%) and GAG (63.9%). Data are presented as mean ± SD.


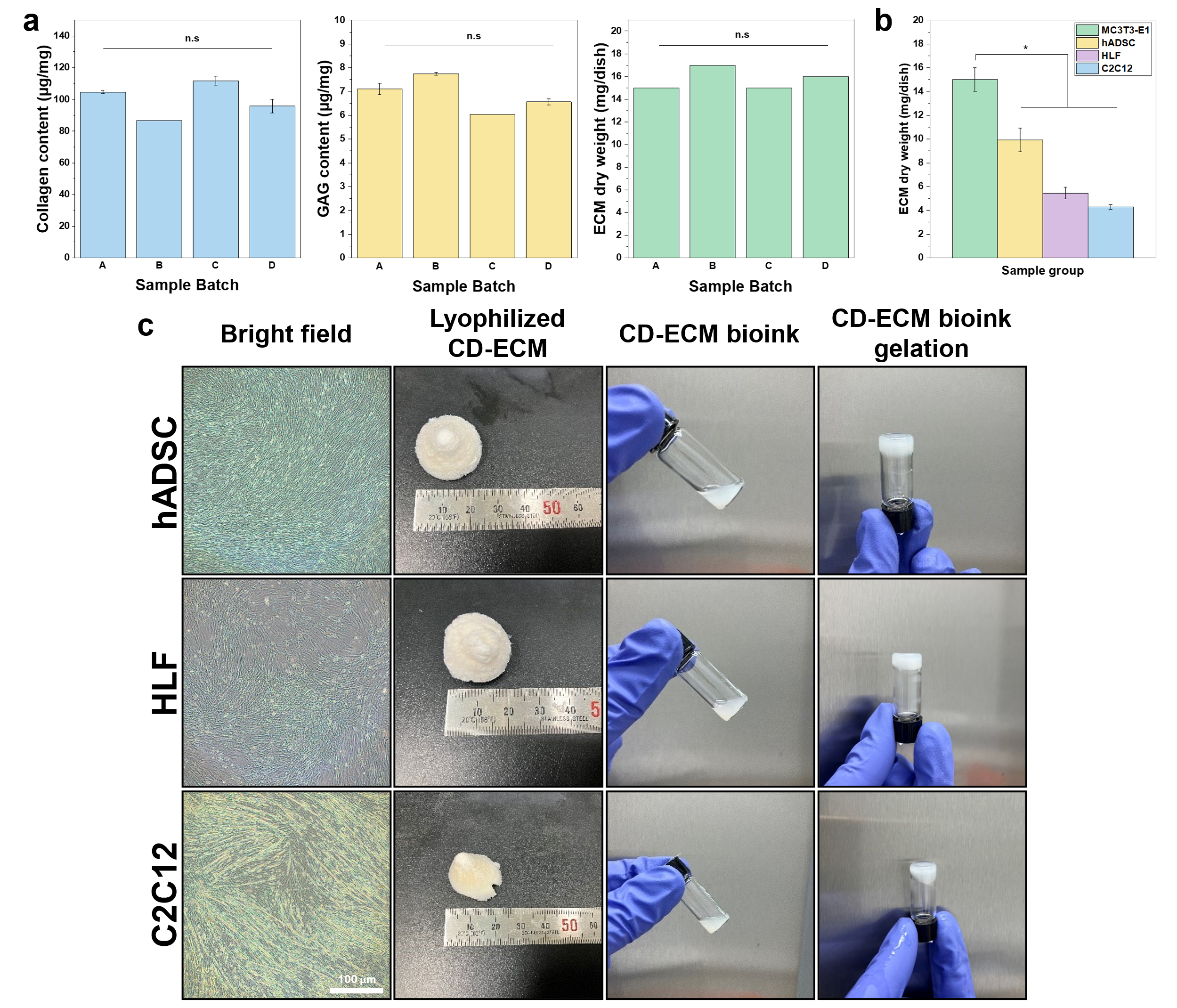


**Supporting Figure S3.** Production consistency and CD-ECM derived from different cell sources. (a) Collagen content, GAG content, and ECM yield among independent CD-ECM sample batches, showing no significant variation (n.s). (b) ECM yield (dry weight per dish) from different cell sources, with MC3T3-E1 producing the highest amount. (c) Representative images of CD-ECM derived from hADSCs, HLFs, and C2C12 cells, shown as cultured cells, freeze-dried ECM, ECM solution, and reconstituted ECM gel. Scale bars: 100 μm. Data are presented as mean ± SD; **p* < 0.05, n.s = not significant.


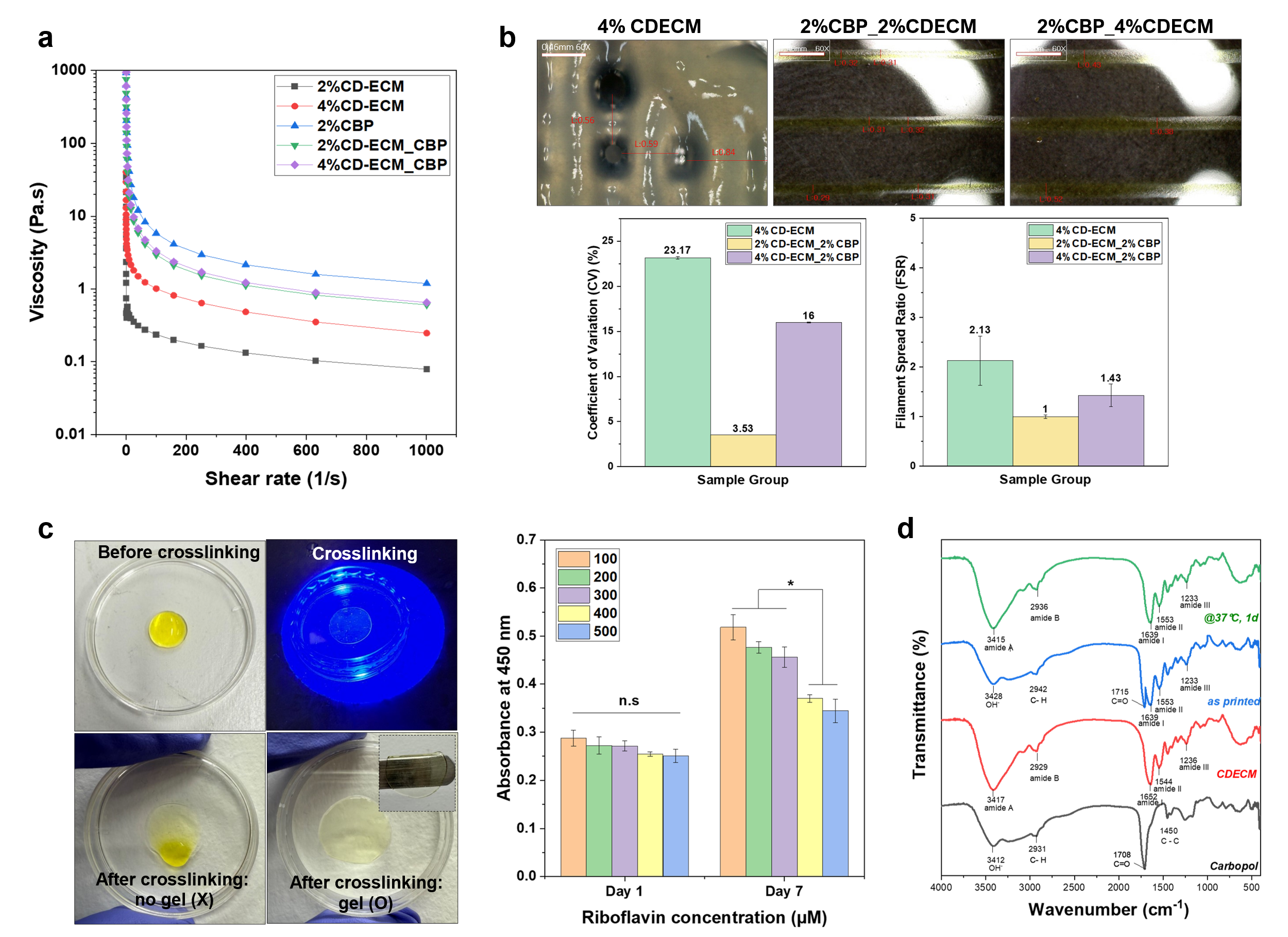


**Supporting Figure S4.** (a) Viscosity analysis demonstrated the shear-thinning behavior of CD-ECM, Carbopol (CBP), and their composite inks. (b) Printability was further assessed by analyzing filament morphology, where the 2% CBP + 2% CD-ECM formulation exhibited the lowest coefficient of variation (CV) and a filament spread ratio (FSR) closest to 1, indicating superior print fidelity. (c) Crosslinking tests using different riboflavin concentrations identified 300 *μ*M RF with 10 mM SPS as the optimal photoinitiator condition, enabling stable gelation while maintaining cell compatibility. Scale bars: 5 mm. (d) Successful removal of CBP after printing was confirmed by FTIR analysis, demonstrating complete elimination of the sacrificial material following incubation in culture medium. Data are presented as mean ± SD; **p* < 0.05, n.s = not significant.


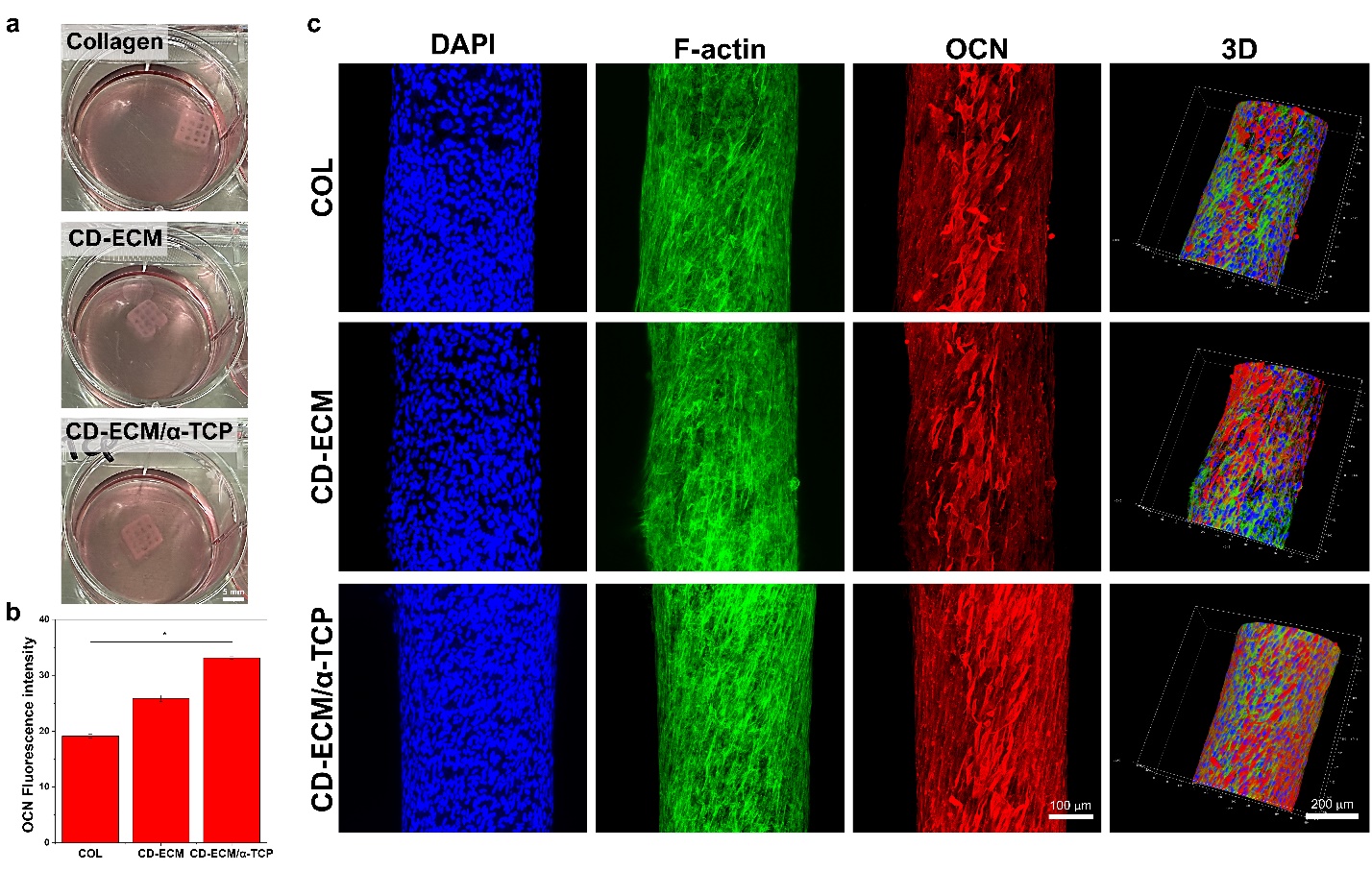


**Supporting Figure S5.** Structural Stability and Immunofluorescence Characterization of Bioprinted Constructs. (a) Structural stability of printed scaffolds during 14-day in vitro culture. Scale bars: 5 mm. (b) Quantitative analysis of OCN fluorescence intensity, demonstrating enhanced osteogenic marker expression and actin organization in the CD-ECM/α-TCP group. (c) Maximum intensity projection of individual immunofluorescence channels of bioprinted constructs showing F-actin (green), osteocalcin (OCN, red), and DAPI (blue) at Week 4. Three-dimensional reconstructed images of the merged signals are also provided. Scale bars: 100 μm (2D) and 200 μm (3D). Data are presented as mean ± SD; *p < 0.05, n.s = not significant.
